# Impact of Sub-Ambient Temperature on Aging Rate and Gas Separation Properties of Polymers of Intrinsic Microporosity

**DOI:** 10.3390/membranes14060132

**Published:** 2024-06-06

**Authors:** Pierre Dieudonné, Riccardo Rea, Elsa Lasseuguette, Maria-Chiara Ferrari

**Affiliations:** Institute for Materials and Processes, University of Edinburgh, Edinburgh EH9 3JL, UK; p.dieudonne@sms.ed.ac.uk (P.D.); rrea2@ed.ac.uk (R.R.); e.lasseuguette@ed.ac.uk (E.L.)

**Keywords:** carbon capture, gas permeation, sub-ambient temperature, glassy polymers, aging, CO_2_/N_2_ separation, self-standing film

## Abstract

Aging in polymers of intrinsic microporosity has slowed exploitation due to a decay in performance over time since densification makes them unsuitable for industrial applications. This work aimed to study the impact of the operation and storage temperature on the gas separation properties and aging rates of PIM-1 self-standing films. The permeability, diffusivity, and solubility of the tested membranes were monitored through permeation tests for pure carbon dioxide and nitrogen at a maximum upstream pressure of 1.3 bar for temperatures ranging from −20 °C to 25 °C. This study found significant benefits in the operation of glassy polymeric membranes at low temperatures, resulting in a favourable trade-off in separation performance and a reduction in the aging rate by three orders of magnitude. This brings new opportunities for the industrial application of PIMs in innovative carbon capture processes.

## 1. Introduction

Polymers of intrinsic microporosity (PIMs) have been studied for almost two decades, showing undeniable potential for gas separation processes. These easily processable materials present very high free volumes of nanopores thanks to their rigid structure, which prevents efficient packing of the membrane matrix [1]. Consequently, PIMs offer high gas permeability (i.e., normalised flux) at relatively high selectivity, which is appealing for gas separation applications and carbon capture and storage (CCS) processes where a large volume of gas is to be treated [2]. Despite these clear advantages, they are yet to be employed in industry primarily due to limitations in stability, with plasticisation and aging negatively impacting CO_2_ selectivity and permeability, respectively [3,4].

The reduction in the free volume with time is a known drawback of glassy polymeric membranes. This known phenomenon translates into a loss of diffusivity (and hence permeability) towards a fixed value at the polymer’s specific volume at equilibrium [5]. The further the material is from its equilibrium, i.e., the larger its excess free volume, the greater impact aging has on its separation properties. As a consequence, aging is especially relevant in high-free-volume polymers such as PIMs [6,7]. Many studies have investigated ways to inhibit this mechanism. In some cases, the polymer structure was determined to be a crucial factor, as ladder-type structures were found to accelerate the densification of glassy polymers [8], while it was found that the addition of fillers in the membrane matrix can be used to slow it down [9,10]. Thicker films are also known to have slower aging rates but are not desired as lower fluxes affect productivity. Additionally, ways to recover the free volume in situ have been investigated, for example, through methanol treatment [11]. Aging is, therefore, another factor to consider when choosing a material for a specific process, in addition to the permeability–selectivity trade-off. The prediction of membrane aging is an important criterion for smooth operability, with designs potentially including previously aged thin films of performant materials from the start to facilitate unit operation.

The operation of membrane modules at low temperatures presents some advantages. Since gas permeation is an activated process, the permeability of the membrane decreases with temperature. However, this is in favour of a more condensable gas, drastically increasing the film selectivity [12]. Furthermore, the storage of a membrane in colder conditions seems to significantly slow its aging. Ji et al. [13] reported that the CO_2_ permeability drop of a PIM-1 membrane was halved after 100 days of aging at 0 °C compared to storage at an ambient temperature. Nonetheless, at present, while systematic investigations have been conducted on the impact of temperature on the aging of glassy polymers [14], studies at sub-ambient temperatures for high-free-volume materials are limited.

The aim of this study was to quantify the impact of a sub-ambient temperature on the aging rate of a high-free-volume polymer, in order to assess the potential for these polymers’ application in CCS processes and their integration with existing cryogenic-based technologies [15]. Specifically, the separation properties of self-standing PIM-1 membranes for carbon dioxide and nitrogen were obtained at different operating temperatures and aging states for post-combustion applications. The results have high relevance since the behaviour of PIM-1 can provide insights into the aging performance of more novel polymers in similar operating conditions.

## 2. Experimental Section

### 2.1. Materials

**Figure 1:**
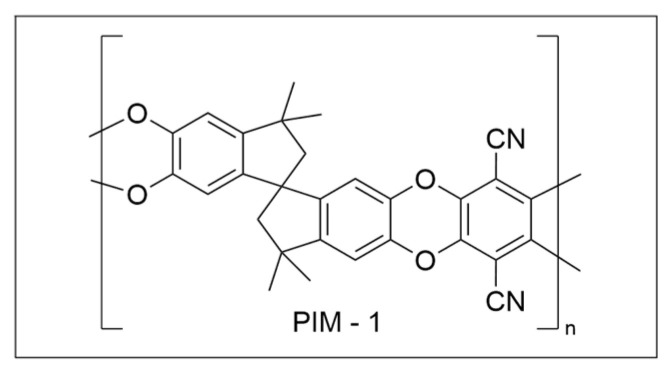
Molecular structure of the PIM-1 utilised in this work.

This study used a single batch of PIM-1 synthesised from the polycondensation of 5,5,6,6-tetrahydroxy-3,3,3,3-tetramethyl-1,1-spirobisindane and tetrafluoroterephtalonitrile, as described by Budd et al. (2005) [16]. The molecular structure of the polymer is shown in Figure 1. Self-standing films were cast from a chloroform (CHCl_3_) solution at 2 wt.% through evaporation at an ambient temperature on a levelled glass Petri dish for about two days. Prior to testing, the PIM-1 membranes were briefly soaked in methanol in order to flush any remaining solvent and reverse any previous aging [17], and then they were left to dry for two hours at an ambient temperature. The membrane thickness was measured just before entering the testing system using a digital micrometre (Mitutoyo, Kawasaki, Japan), consistently obtaining a thickness of ~45 µm. The obtained membranes were left overnight under a dynamic vacuum to be tested the following morning.

### 2.2. Methods

**Figure 2:**
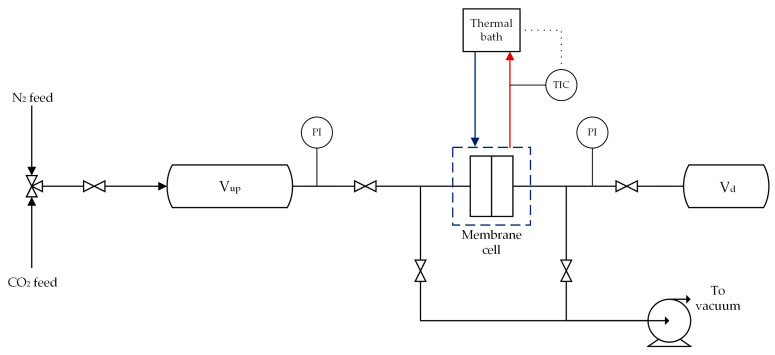
Schematic of constant-volume variable-pressure permeation apparatus used in this work.

The membranes’ gas separation properties were measured using the constant-volume variable-pressure permeation apparatus presented in Figure 2, with an operating range from −40 °C to 100 °C. Temperature control was implemented using a thermal jacket around the membrane cell cooled by a refrigerated bath (Julabo GmbH, Seelbach, Germany). The gas temperature was calibrated against the thermal bath temperature to guarantee consistent testing conditions. Additionally, the downstream volume temperature was measured using a thermocouple during each experiment (British Rototherm Company Ltd., Port Talbot, UK).

Permeability, diffusivity, and solubility were obtained by measuring the build-up of downstream pressure of pure gas permeating through a membrane of surface area 2.9 cm^2^. The upstream pressure was fixed at 1.3 bar and the operating temperature range for this study was from −20 °C to 25 °C. Nitrogen was always tested before carbon dioxide to avoid any potential plasticisation in the membrane. Downstream pressures were recorded using a capacitance manometer (Brooks Instrument, Hatfield, PA, USA) with a maximum scale of 1.0 bar and accuracy of 0.25% of the reading. Leakage in the permeation cell and downstream volume was limited to a maximum of 1% of the total permeation rate.

When tested for short-term aging, the membranes were left in the membrane cell at the temperature of the test under a static vacuum. The polymer powder and cast films were stored at an ambient temperature in sealed packages when out of the system.

Gas separation properties can be obtained using the time-lag method [18]. Permeability *P* (in Barrer) and diffusivity *D* (in cm^2^/s) were calculated using the following equations:

(1) $$P=10^{10}\times \frac{V_{d}\;l}{p_{up}\;A\;R\;T_{d}}\times \left[{\left(\frac{dp_{d}}{dt}\right)}_{ss}-{\left(\frac{dp_{d}}{dt}\right)}_{leak}\right]$$

(2) $$D=l^{2}/6\theta$$

with $V_{d}$ as the downstream volume (in cm^3^), l as the membrane thickness (in cm), $p_{up}$ as the initial upstream pressure (in cmHg), A as the membrane surface area exposed to gas (in cm^2^), R as the gas constant (0.278 cm^3^.cmHg/cm^3^(STP).K), $T_{d}$ as the downstream temperature (in K), and θ as the time lag in permeation measurement (in s). The terms ${\left(dp_{d}/dt\right)}_{ss}$ and ${\left(dp_{d}/dt\right)}_{leak},$ respectively, represent the downstream pressure build-up (in cmHg/s) in a steady state during operation and due to a system leak.

The solubility coefficient S (in cm^3^(STP)/cm^3^.cmHg) was determined from

(3) S=P/D

The diffusivity value was confirmed using full permeation curve fitting. Assuming no initial gas concentration in the membrane, fitting was performed according to the following expression of diffusivity through a plane sheet, as derived by Crank [19]:

(4) $$Q_{t}=D\left(C_{up}-C_{d}\right)\frac{t}{l}+\frac{2l}{\pi^{2}}\sum_{n=1}^{\infty }\frac{C_{up}\cos n\pi -C_{d}}{n^{2}}\left\{1-\exp\left(-Dn^{2}\pi^{2}t/l^{2}\right)\right\}$$

where $Q_{t}$ is the total amount of diffusing substance in the downstream volume (in cm^3^ (STP)) at time t (in s), and $C_{up}$ and $C_{d}$ are the gas concentration in the membrane at the gas/membrane interface upstream and downstream, respectively (in cm^3^ (STP)/cm^3^).

Ideal selectivity $\alpha_{i/j}$ is defined as the ratio of the permeabilities of pure gases i and j as follows:

(5) $$\alpha_{i/j}=P_{i}/P_{j}$$

Pure CO_2_ sorption isotherms for the PIM-1_D membrane (about 275 mg) were measured by means of a high-pressure adsorption differential volumetric apparatus (ADVA-60), entirely designed at the University of Edinburgh. The differential apparatus is composed of two parallel and symmetrical branches, one containing the sample while the other is used as a reference. The system presents a very similar design to the low-pressure ADVA-1 [20]. ADVA-60 is equipped with 2 absolute pressure transducers with full ranges of 2 and 60 bar, respectively, and a differential sensor, placed in between the two branches, with a full-scale reading of ±350 mbar. The transducers’ accuracy is 0.04% of the full scale (Baker-Hughes Company UNIK-5000 series). Four thermocouples (Omega Engineering TJFT72 series) are inserted in the dosing and uptake volumes of ADVA-60, one for each, for the direct measurement of the gas temperature. All the valves are fast-acting pneumatic actuated valves (Swagelok HB series).

While the dosing section is exposed to room temperature, the uptake part is immersed in a circulating liquid bath served by an external chiller (Julabo Corio CD-200 series). Measurements were performed in a stepwise manner up to a maximum of 1.7 bar. The equilibrium experiment was repeated 5 times over a week in the same temperature and pressure conditions. The sample was outgassed overnight in situ at the temperature of the test. For that purpose, the HiCube 80 vacuum station (Pfeiffer Vacuum) connected to the system guaranteed a high level of vacuuming for complete sample outgassing.

The resulting equilibrium data were fitted according to the dual sorption model as follows [21]:

(6) $$C=k_{D}p+\frac{C_{H}^{\prime }bp}{1+bp}$$

with C as the concentration of the gas in the membrane (in cm^3^(STP)/cm^3^), $k_{D}$ as the Henry constant (in cm^3^(STP)/cm^3^.cmHg) for the linear contribution, p as the pressure (in cmHg), and $C_{H}^{\prime }$ (in cm^3^(STP)/cm^3^) and b (in cmHg^−1^) as the Langmuir saturation capacity and affinity parameters, respectively.

The temperature dependences of permeability, diffusivity, and solubility in polymeric membranes are typically described by the Arrhenius–van’t Hoff model [22], assuming no change in material structure in the studied temperature range, respectively:

(7) $$P=P_{0}\times \exp\left(-\frac{E_{P}}{RT}\right)$$

(8) $$D=D_{0}\times \exp\left(-\frac{E_{D}}{RT}\right)$$

(9) $$S=S_{0}\times \exp\left(-\frac{∆H}{RT}\right)$$

where $E_{P}$ is the activation energy of permeability, $E_{D}$ is the activation energy of diffusivity, and ∆H is the heat of sorption of the gas in the membrane (in kJ/mol). $P_{0}$, $D_{0}$, and $S_{0}$ are pre-exponential factors of units corresponding to permeability, diffusivity, and solubility, respectively.

## 3. Results and Discussion

### 3.1. PIM-1 Separation Properties at Low Temperatures

**Figure 3:**
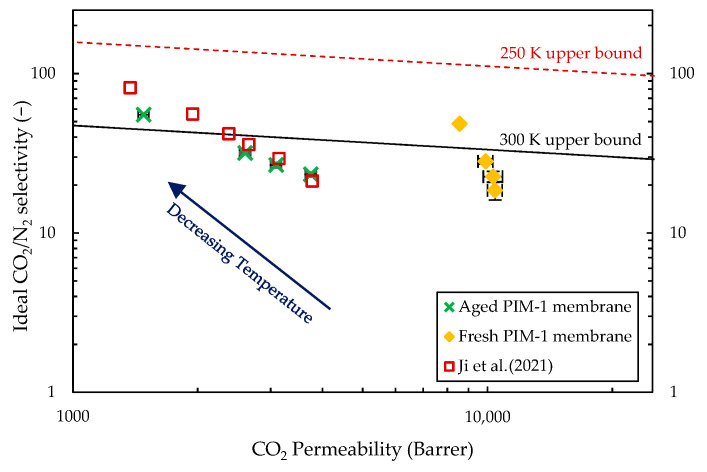
Robeson plot presenting fresh and aged (500 days) PIM-1_A films tested in this study compared to a PIM-1 membrane from [13] tested between 30 °C and −30 °C. Tests at all temperatures were performed in about 24 h to limit the aging time.

The separation properties of a PIM-1 membrane, PIM-1_A, were determined at different aging states. The fresh and aged membranes, as expected, showed significant difference in permeability, with CO_2_ permeability dropping from 10,000 Barrer to 3500 Barrer at 25 °C after 500 days, as can be seen in Figure 3.

A large drop in permeability of CO_2_ and N_2_ was also observed when decreasing the operating temperature in both aging stages (Figure 3). The fresh membrane had its CO_2_ permeability drop by 46% from 25 °C to −20 °C, while that decrease reached 60% for the aged membrane. Nitrogen transport properties were impacted to a larger extent, tripling the ideal CO_2_/N_2_ selectivity and offering overall a beneficial performance trade-off. Tests at low temperatures thus managed to overcome the 2008 upper bound defined at 30 °C, but remained below it when taking into account the change in temperature [23] since the membrane position relative to the upper bound is material dependent.

Table 1 shows that aging increased the activation energy of diffusivity and hence permeability. In fact, 500 days of aging at an ambient temperature caused the activation energy of CO_2_ diffusivity of the membrane to increase by 75%. This is due to the collapse of the micropores with time, leaving the diffusion of gas through the membrane matrix increasingly dependent on the gas particle frequency of movement in the membrane matrix, and hence on temperature [24]. While the activation energy of permeability for nitrogen was quite close to that found in the literature for an aged membrane, values for the diffusivity and heat of sorption could not be determined due to the large uncertainty in time-lag measurement at a relatively low permeability.

In addition to matching the general trend of permeation results with decreasing temperature, the aged membrane activation energies were found to be similar to the work from Ji, W. et al. [13], which used a fresh membrane not treated with methanol. In particular, this was the case for CO_2_. The results obtained for the fresh membrane fell within the values found in the literature for PIM-1 [25,26,27].

### 3.2. Study of Aging in PIM-1

#### 3.2.1. Impact of Aging on Gas Separation Properties

The change in PIM-1 membranes’ separation properties with aging was studied in three samples presenting comparable initial diffusivities at 10 °C. PIM-1_B and PIM-1_C were prepared as indicated in Section 2.1 while PIM-1_D was deliberately made thicker (~63 µm).

**Figure 4:**
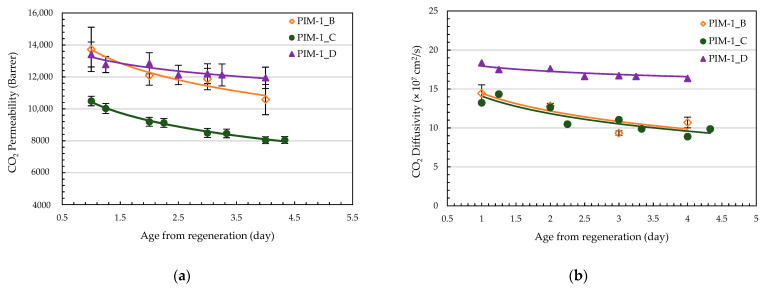
CO_2_ (**a**) permeability and (**b**) diffusivity aging trends of three membranes stored and operated at 10 °C. The full lines are guides for the eyes.

The trends in the decreases with time of permeability and diffusivity (Figure 4a and 4b, respectively) were similar for the three membranes tested. In particular, PIM-1_B and PIM-1_C had overlapping diffusivity and comparable permeability aging trends despite different initial P_CO2_ values, at 10,500 Barrer and 13,700 Barrer, respectively. In comparison, PIM-1_D showed similar initial transport properties to PIM-1_B. However, its aging was much less pronounced, which could be inferred as resulting from its greater thickness slowing down film densification [28]. This indicates that the aging is only diffusivity (i.e., free volume) dependent, and, therefore, solubility should not be impacted by aging, which is consistent with observations from the literature [29,30]. This was verified by comparing the solubility of PIM-1_D obtained from both volumetric direct measurements and indirect calculation from permeation experiments.

**Figure 5:**
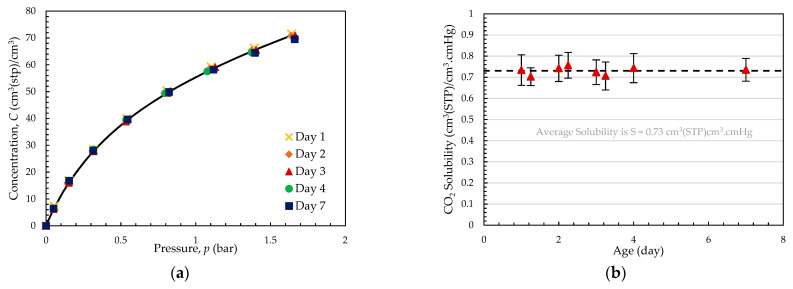
(**a**) CO_2_ sorption isotherms with fitting line obtained from Equation (6) and (**b**) solubility obtained from permeation test of PIM-1_D at 10 °C.

Figure 5a shows the sorption isotherms obtained during a week of testing. The curves overlap and were fitted using Equation (6) with sorption parameters of $k_{D}=0.18$ cm^3^(STP)/cm^3^.cmHg, $C_{H}^{\prime }=64.65$ cm^3^(STP)/cm^3^, and $b=2.51\times 10^{-2}$ cmHg^−1^. In the conditions used in the permeation test, the solubility of CO_2_ would be $S_{CO_{2}}=0.70$ cm^3^(STP)/cm^3^.cmHg, which is in good agreement with the average values obtained in the permeation experiments, shown in Figure 5b.

Negligible deviations of solubility were observed over the first week from regeneration of the membrane for both methods, as shown in Figure 5, confirming that the impact aging has on permeability arises exclusively from the change in membrane diffusivity. Furthermore, aging has no impact on the temperature dependence of solubility, as evident in Table 1 when comparing the heat of sorption of CO_2_ in the fresh and aged PIM-1_A membranes.

**Figure 6:**
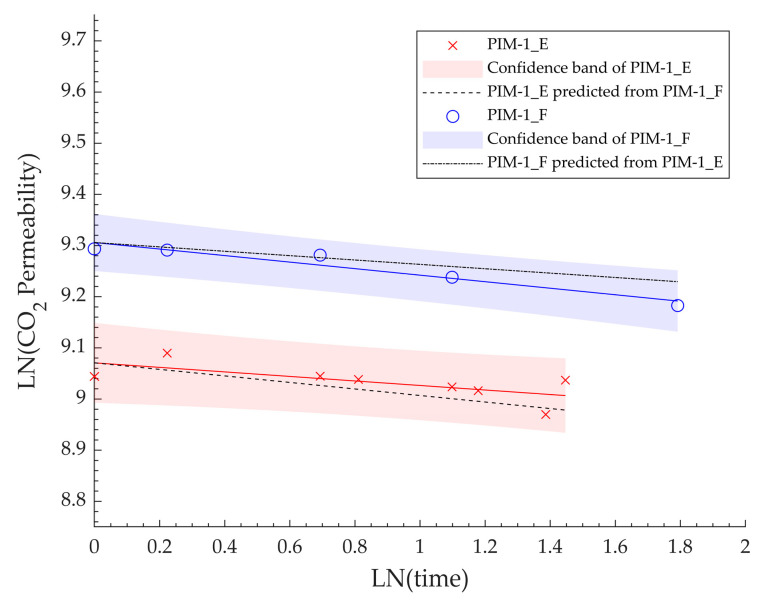
Experimental and predicted aging trendlines of two PIM-1 membranes tested at 0 °C.

These results highlight that the degree of aging of PIM-1 at a certain temperature and pressure can be predicted given its initial diffusivity or permeability and the full aging curve of any membrane made from the same material. This was further confirmed at different temperature using two membranes, PIM-1_E and PIM-1_F, presenting similar initial CO_2_ diffusivity (1.35 ± 0.15 × 10^−6^ cm^2^/s). The change in their permeability with aging at 0 °C is presented in Figure 6.

The experimental data were fitted linearly on a log–log scale to obtain the membranes’ aging rates, expressed as the unitless constant $\beta_{P}=-d(\ln P_{CO_{2}})/d(\ln t)$, with the bands displayed on the graph showing the 95% confidence interval in the trendline. Both membranes, as expected, presented close aging rates, at 4.42 × 10^−2^ and 6.38 × 10^−2^ for PIM-1_E and PIM-1_F, respectively. The aging rate of one membrane was used to predict the aging behaviour of the other from their initial permeability. The resulting lines fell within the confidence interval despite moderately (~20%) different initial diffusivity values, showing that the prediction of a membrane aging is indeed possible given material aging data in the same operating conditions.

#### 3.2.2. Effect of Temperature on Aging Rate

**Figure 7:**
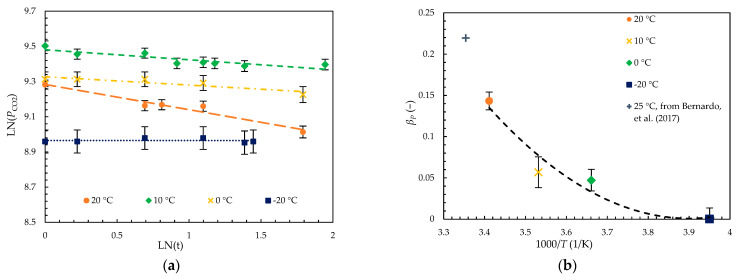
Change in (**a**) permeability $P_{CO_{2}}$ over the first week of aging and (**b**) aging rate $\beta_{P}$ in four PIM-1 membranes stored and operated from 20 °C to −20 °C. Dashed lines in graph (**a**) represent the linear trendline used to calculate the aging rates while the curved line in (**b**) represents the general trendline of $\beta_{P}$ with decreasing temperature. Bernardo, et al. (2017) permeation data were measured at 25 °C [31].

Membranes of comparable initial permeabilities were stored and operated at different temperatures for a week. As expected, their aging rates $\beta_{p}$ were greatly influenced by temperature, as can be observed in Figure 7. Variations in initial permeability arose from the difference in operating temperature as well as the difference in initial diffusivity, i.e., free volume. The discrepancies in the initial membrane free volume were likely due to variations in casting conditions, but were deemed to have minimal impact on the observed trendlines as they fell within the displayed error, as discussed previously.

A clear trend could be observed where membranes stored at lower temperatures saw a significant decrease in their aging rates. Indeed, $\beta_{P}$ dropped by three orders of magnitude from 20 °C to −20 °C, from 1.37 × 10^−1^ down to 2.08 × 10^−4^. Thus, short-term aging seems to be effectively inhibited at a sub-ambient temperature. Beyond this, while it seems as though material relaxation can be completely stopped by further decreasing temperature, the change in aging rate at cryogenic temperatures would become minimal and would not justify the extra cooling required.

Finally, it was also observed that the storage of several self-standing films in a low-temperature environment (−20 °C) did not impact their structural integrity. These findings are important in the context of both research and industry. Keeping membranes in a cold environment prior to or in between experiments can ensure that the following test will not be impacted by aging. This presents an opportunity for repeatability studies or comparative tests, to remove the unknown aging factor from the results. In practical applications, processes including membrane modules operating at a sub-ambient temperature have been proposed [32]. Combined with the development of aging-resistant high-performance polymers [33], the operation of gas separation membranes in cryogenic conditions brings opportunities for the use of high-free-volume polymeric membranes in industrial processes, where their application is currently impeded by high aging rates. 

## 4. Conclusions

This study has shown the significant impact of the operating and storage temperatures of PIM-1 membranes on their gas separation properties and stability. Permeation tests in sub-ambient conditions revealed a favourable performance trade-off from 20 °C to −20 °C, boosting the selectivity by threefold at the expense of some of the CO_2_ permeability. This investigation confirmed previous observations on the aging of high-free-volume polymeric membranes. It showed a drastic decrease in the diffusivity value, and an increase in its temperature dependence with time. Moreover, a membrane aged at an ambient temperature for 500+ days had its activation energy of diffusivity increased by 75% while its heat of sorption remained unchanged. In addition, both the volumetric and permeation methods showed a constant solubility in a PIM-1 membrane operated at 10 °C over a week, revealing no apparent impact of aging on solubility. Thus, it seems possible to predict aging knowing only a material’s initial diffusivity and its aging rate, which can be advantageous in industry for better control in operations. Finally, tests conducted for a week at constant temperatures showed a significant inhibition of aging in sub-ambient conditions, reducing the aging rate of a high-free-volume polymeric membrane by three orders of magnitude from 20 °C to −20 °C, bringing new opportunities for research and industrial applications.

## Figures and Tables

**Table 1:** Permeability and diffusivity activation energies and heat of sorption of the fresh and aged PIM-1_A membrane shown in Figure 3.

		E_P_	E_D_	ΔH
		kJ/mol	kJ/mol	kJ/mol
Fresh PIM-1_A (day 1–3)	CO_2_	2.88	15.56	−12.68
	N_2_	16.52	30.89	−14.37
Aged PIM-1_A (day 500+)	CO_2_	12.75	25.09	−12.34
	N_2_	25.31	ND	ND
Ji et al. (2021) [13]	CO_2_	11.88	25.44	−15.40
	N_2_	23.01	37.66	−14.10
Li et al. (2014) [25]	CO_2_	1.70	17.60	−15.90
	N_2_	11.90	25.10	−13.00
Budd et al. (2008) [26]	CO_2_	−1.50	-	-
	N_2_	11.90	-	-
Thomas et al. (2009) [27]	CO_2_	−1.00	-	-
	N_2_	14.30	-	-

## Data Availability

The original data presented in this study are openly available at https://doi.org/10.7488/ds/7737 (accessed on 4 June 2024).
